# S-1-Based versus Capecitabine-Based Preoperative Chemoradiotherapy in the Treatment of Locally Advanced Rectal Cancer: A Matched-Pair Analysis

**DOI:** 10.1371/journal.pone.0106162

**Published:** 2014-09-02

**Authors:** Meng Su, Lu-Cheng Zhu, Hang-Ping Wei, Wen-Hua Luo, Rui-Fang Lin, Chang-Lin Zou

**Affiliations:** Department of Radiation Oncology and Chemotherapy, The First Affiliated Hospital of Wenzhou Medical University, Wenzhou, China; University General Hospital of Heraklion and Laboratory of Tumor Cell Biology, School of Medicine, University of Crete, Greece

## Abstract

**Objective:**

The aim of this paper was to compare the efficacy and safety of S-1-based and capecitabine-based preoperative chemoradiotherapy regimens in patients with locally advanced rectal cancer through a retrospective matched-pair analysis.

**Materials and methods:**

Between Jan 2010 and Mar 2014, 24 patients with locally advanced rectal cancer who received preoperative radiotherapy concurrently with S-1 were individually matched with 24 contemporary patients with locally advanced rectal cancer who received preoperative radiotherapy concurrently with capecitabine according to clinical stage (as determined by pelvic magnetic resonance imaging and computed tomography) and age (within five years). All these patients performed mesorectal excision 4–8 weeks after the completion of chemoradiotherapy.

**Results:**

The tumor volume reduction rates were 55.9±15.1% in the S-1 group and 53.8±16.0% in the capecitabine group (p = 0.619). The overall downstaging, including both T downstaging and N downstaging, occurred in 83.3% of the S-1 group and 70.8% of the capecitabine group (*p* = 0.508). The significant tumor regression, including regression grade I and II, occurred in 33.3% of S-1 patients and 25.0% of capecitabine patients (*p* = 0.754). In the two groups, Grade 4 adverse events were not observed and Grade 3 consisted of only two cases of diarrhea, and no patient suffered hematologic adverse event of Grade 2 or higher. However, the incidence of diarrhea (62.5% *vs* 33.3%, *p* = 0.014) and hand-foot syndrome (29.2% *vs* 0%, *p* = 0.016) were higher in capecitabine group. Other adverse events did not differ significantly between two groups.

**Conclusions:**

The two preoperative chemoradiotherapy regimens were effective and safe for patients of locally advanced rectal cancer, but regimen with S-1 exhibited a lower incidence of adverse events.

## Introduction

Neoadjuvant therapy for locally advanced rectal cancer (LARC) has gained widespread acceptance today. Preoperative radiotherapy (RT) significantly reduced the risk of local recurrence and death from rectal cancer, and the addition of fluoropyrimidine provided further benefits in local control [1]–[4]. Therefore, fluorouracil-based preoperative chemoradiotherapy (CRT) becomes one of the standard therapy for LARC and oral fluoropyrimidine has gradually taken the place of continuous 5-FU infusion because of its convenience and safety [5].

Capecitabine (Xeloda; Hoffman-La Roche Ltd, Basel, Switzerland) is an oral fluoropyrimidine anticancer agent with substantial activity in colorectal cancer and it has been frequently used in preoperative CRT [6], [7]. But diarrhea and hand-foot syndrome were inevitable for mostly patients who used capecitabine.

S-1 (TS-1; Taiho Pharmaceutical, Tokyo, Japan) is another oral fluoropyrimidine anticancer agent, which had similar, and in some cases superior activity, to other active chemotherapies for the treatment of colorectal cancer patients with a promising safety profile [8]. And recently S-1 was applied to preoperative CRT for patients with LARC, which revealed a high efficacy and low incidence of adverse events [9].

Although lots of papers have demonstrated that both oral fluoropyrimidines were effective and safe, doubts still exist about whether S-1 or capecitabine is the better choice when associated with preoperative RT for patients with LARC. Based on this consideration, the aim of this study was to compare the efficacy and safety of S-1-based and capecitabine-based preoperative CRT regimens in patients with LARC through a retrospective matched-pair analysis.

## Materials and Methods

### Patients

From Jan 2010 to Mar 2014, the medical records of rectal cancer patients were retrospectively reviewed in our hospital. 24 patients with histopathologically confirmed as rectal adenocarcinoma, clinically staged as T3–T4, Tx N+, M0, no history of anticancer therapy, who received preoperative RT concurrently with S-1, were included in this study. For comparison, these patients were individually matched with 24 contemporary patients with histopathologically confirmed as rectal adenocarcinoma, clinically staged as T3–T4, Tx N+, M0, no history of anticancer therapy, who received preoperative RT concurrently with capecitabine. Matching criteria were clinical stage (as determined by pelvic magnetic resonance imaging and computed tomography) and age (within five years). Matching by clinical T stage and N stage was not done in this study because it would have significantly reduced the sample size.

### Ethics

This study was approved by the Ethics Committee of the First Affiliated Hospital of Wenzhou Medical University, Wenzhou, China. Written informed consents were obtained from all the patients for publication of this report.

### Chemotherapy

Preoperative chemotherapy was delivered throughout the period of pelvic radiation. S-1 and capecitabine were respectively given twice a day in a fixed daily dose of 80 mg/m2 and 825 mg/m2 on days 1 to 14, 22 to 35. The choice of S-1 or capecitabine was determined by the preferences of patients or physicians.

### Radiotherapy

The total irradiation dose of 50 Gy was delivered in daily fractions of 2 Gy, five times per week, through a pair of opposed anterior-posterior fields using a 6 MV linear accelerator. The treatment fields were set as follows: The superior border was placed at S1/L5, the inferior border was placed at 3–4 cm below the lowest tumor border or the inferior margin of the obturator foramen, and the lateral borders of the planning target volume were 1.5 cm lateral to the widest bony margin of the true pelvic wall.

### Surgery

Surgery was performed 4–8 weeks after the completion of CRT. The total mesorectal excision was the main surgical treatment, and the final choice of low anterior resection or abdominoperineal resection was determined by estimation of surgeon and the will of patient.

### Assessment

Tumor responses, including clinical response and pathological response, were evaluated in this paper. The clinical response was determined by assessing the degree of tumor shrinkage. As two assessment instruments of clinical tumor response, magnetic resonance imaging and computed tomography were performed before CRT and 2–7 days before surgery. Tumor volume measurement was based on the Response Evaluation Criteria in Solid Tumors (RECIST) [10].

The pathological responses, including downstaging and tumor regression, were evaluated by examining the resected specimens. Downstaging was determined by comparing the pretreatment clinical stage with the postoperative pathological stage, and the overall downstaging included both T downstaging and N downstaging. Tumor stage was defined according to the 7^th^ edition of the American Joint Committee on Cancer staging manual [11]. The tumor regression grade (TRG) was classified in 5 levels: TRG I (pathological complete response); TRG II (rare residual cancer cells); TGR III (fibrosis outgrowing residual cancer); TGR IV (residual cancer cells outgrowing fibrosis); TGR V (absence of regressive changes) [12]. We defined significant tumor regression (STR) as TRG I/II.

Safety was analyzed by assessing the incidences of adverse events. The adverse event grades were defined according to the Common Terminology Criteria for Adverse Events of the National Cancer Institute, version 3.0 [13].

### Statistical analysis

In this matched-pair study, McNemar's chi-square test or Fisher's exact test was used to compare the categorical variables, and the paired *t* test or Wilcoxon signed rank test was used to compare the continuous variables. The Wilcoxon signed rank test was also used to compare the rates of adverse events between the two groups, taking into account the different adverse event grades. Statistical analyses were performed with the Statistical Package for Social Sciences, version 17.0 (SPSS Inc., Chicago, IL, USA). Differences with *p*<0.05 were considered to indicate statistical significance and all statistical tests were two sided.

## Result

### Patients

24 patients with LARC who received preoperative RT concurrently with S-1 were matched by clinical stage and age with 24 patients with LARC who received preoperative RT concurrently with capecitabine. The baseline characteristics of the patients of two groups were recorded and are showed in Table 1. There was no significant differences between two groups for gender, distance from anal verge, Eastern Cooperative Oncology Group (ECOG) performance states (PS), clinical T classification, clinical N classification, histological differentiation, pre-CRT carcinoembryonic antigen (CEA).

**Table 1:** Patients' characteristics.

Characteristics	S-1 (n = 24)	Capecitabine (n = 24)	*p*-value
**Gender (%)**			1.000
Male	19 (79.2)	18 (75.0)	
Female	5 (20.8)	6 (25.0)	
**Age (years)**			Matched
Mean (SD)	54.8 (10.0)	55.4 (9.8)	
Median (range)	54 (33–80)	54.5 (35–83)	
**Distance from anal verge (cm)**			0.503
Mean (SD)	5.1 (2.2)	5.5 (1.9)	
Median (range)	5.0 (0.5–10)	6.0 (1–8)	
**ECOG PS (%)**			0.754
0	14 (58.3)	12 (50.0)	
1	10 (41.7)	12 (50.0)	
**Clinical T classification (%)**			0.727
cT3	12 (50.0)	14 (58.3)	
cT4	12 (50.0)	10 (41.7)	
**Clinical N classification (%)**			1.000
cN0	7 (29.2)	7 (29.2)	
cN+	17 (70.8)	17 (70.8)	
**Clinical stage (%)**			Matched
cStage II	7 (29.2)	7 (29.2)	
cStage III	17 (70.8)	17 (70.8)	
**Histological differentiation (%)**			0.667
Poorly	5 (20.8)	6 (25.0)	
Moderately	16 (66.7)	13 (54.2)	
Well	3 (12.5)	5 (20.8)	
**Pre-CRT CEA (ng/ml) (%)**			1.000
≤5	15 (62.5)	14 (58.3)	
>5	9 (37.5)	10 (41.7)	

*ECOG PS* Eastern Cooperative Oncology Group performance states; *CRT* chemoradiotherapy; *CEA* carcinoembryonic antigen.

### Efficacy

The clinical response and pathological response of two groups are presented in Table 2. The tumor volume reduction rates were 55.9±15.1% (mean ± SD) in S-1 group and 53.8±16.0% in capecitabine group (*p* = 0.619). T downstaging rates were 62.5% for S-1 group and 50.0% for capecitabine group (*p* = 0.549). 70.6% of cN+ patients in S-1 group and 58.8% of cN+ patients in capecitabine group changed into pN- (*p* = 0.687). In S-1 group and capecitabine group, overall downstaging (including T downstaging and N downstaging) rates were 83.3% and 70.8% respectively (*p* = 0.508), and STR (including regression grade I and II) occurred in 33.3% of S-1 patients and 25.0% of capecitabine patients (*p* = 0.754).

**Table 2:** Clinical and pathologic evaluations of tumor response.

	S-1 (n = 24)	Capecitabine (n = 24)	*p*-value
**Tumor shrinkage (%)**			0.619
Mean (SD)	55.9 (15.1)	53.8 (16.0)	
**Tumor downstage (%)**			
T downstaging	15 (62.5)	12(50.0)	0.549
N downstaging*	12 (70.6)	10 (58.8)	0.687
Overall downstaging	20 (83.3)	17 (70.8)	0.508
**Tumor regression grade (TRG) (%)**			
TRG I/II (STR)	8 (33.3)	6 (25.0)	0.754
TRG III/IV	14 (58.3)	17 (70.8)	0.508
TRG V	2 (8.3)	1 (4.2)	1.000

*STR* significant tumor regression.

*17 patients in S-1 group and 17 patients in capecitabine group were demonstrated as cN+.

### Safety

Table 3 presents the treatment-related adverse events which observed among all 48 patients during the period of preoperative CRT. Hand-foot syndrome was only observed in capecitabine group (29.2% *vs* 0%, *p* = 0.016). The incidence of diarrhea was also higher in capecitabine group (62.5% *vs* 33.3%, *p* = 0.014). Except diarrhea and hand-foot syndrome, adverse events did not differ significantly between two groups. Grade 4 adverse events did not occur in either of the groups, and no Grade 2 or higher hematologic adverse event was observed. Besides, only two patients experienced Grade 3 adverse events of diarrhea. Adverse events were mild in two groups, and most of them were relieved after appropriate treatment.

**Table 3:** Adverse event profiles during treatment.

	S-1 (n = 24)		Capecitabine (n = 24)		*p*-value
	Total (%)	Grade 1/2/3	Total (%)	Grade 1/2/3	
**Hematologic**					
Leukopenia	5 (20.8)	5/0/0	4 (16.7)	4/0/0	1.000
Neutropenia	4 (16.7)	4/0/0	2 (8.3)	2/0/0	0.688
Anemia	1 (4.2)	1/0/0	1 (4.2)	1/0/0	1.000
Thrombocytopenia	2 (8.3)	2/0/0	0 (0)	0/0/0	0.500
**Non-hematologic**					
Diarrhea	8 (33.3)	6/2/0	15 (62.5)	7/6/2	0.014
Vomiting	5 (20.8)	4/1/0	9 (37.5)	7/2/0	0.340
AST/ALT	1 (4.2)	1/0/0	1 (4.2)	1/0/0	1.000
Hand-foot syndrome	0 (0)	0/0/0	7 (29.2)	5/2/0	0.016

*AST* aspartate aminotransferase; *ALT* alanine transaminase.

### Surgery

The clinical stage after CRT are showed in Table 4 and did not differ significantly between two groups. All 48 patients underwent radical surgery 4–8 weeks after the completion of chemoradiation. Among them, 38 patients (79.2%) received low anterior resection and 10 patients (20.8%) received abdominoperineal resection. Of the 25 patients who had rectal cancer within 5 cm of the anal verge, 18 patients (72.0%) underwent sphincter preserving surgery. All the patients (100%) had a negative circumferential resection margin.

**Table 4:** Clinical stage after chemoradiotherapy.

	S-1 (n = 24)	Capecitabine (n = 24)	*p*-value
**Clinical T classification (%)**			0.556
cT1–2	11 (45.8)	8 (33.3)	
cT3–4	13 (54.2)	16 (66.7)	
**Clinical N classification (%)**			0.740
cN0	19 (79.2)	17 (70.8)	
cN+	5 (20.8)	7 (29.2)	
**Clinical stage (%)**			0.666
cStage I	9 (37.5)	6 (25.0)	
cStage II	10 (41.7)	11 (45.8)	
cStage III	5 (20.8)	7 (29.2)	

### Postoperative

After surgery, only 1 patient in capecitabine group who received abdominoperineal resection suffered from massive intestinal bleeding and died one month later. No other serious postoperative complications occurred. Of the patients, 47 received fluoropyrimidine-based adjuvant chemotherapy (capecitabine, S-1, FOLFOX or XELOX). Until now, no local recurrence was observed and distant metastasis was only observed in 2 patients of capecitabine group (1 liver metastases, 1 liver and lung metastases). Except 1 patient died of massive intestinal bleeding, all the patients are alive.

## Discussion

The short-term aim of treatment for rectal cancer is to achieve complete resection of the tumor, and the long-term aims are to improve overall survival and disease free survival through a high locoregional control and low distant metastasis rate. However, local advanced ones are difficult to reach these goals if receive surgery alone, hence neoadjuvant therapy followed by radical surgery has been introduced to enhance the outcome of LARC [14]. In addition, the neoadjuvant therapy does not increase perioperative complications [15]. According to previous clinical studies, preoperative CRT is superior to preoperative RT by improving pathological response and local control [2]–[4], [16], and it could provide advantages of local control, toxicity, compliance and sphincter preservation rate when compares with postoperative approach [17]. Sphincter preservation is important for LARC patients to preserve a high quality of life.

For years, 5-FU as an essential role in the treatment of rectal cancer has been widely used in CRT. As a radiosensitizer in preoperative CRT, 5-FU usually administered as protracted intravenous infusions. However, protracted intravenous infusions of 5-FU are inconvenient, and patients with central venous catheters are associated with a non-negligible risk of complications such as infection and thrombosis. Nowadays, the quality of life for cancer patients is increasingly concerned. Therefore, oral fluoropyrimidines such as capecitabine and S-1 have been developed to take the place of intravenous 5-FU. Capecitabine and S-1 are well tolerated which mimic continuous-infusion 5-FU, whilst promising improve patient convenience and quality of life [5], [8].

Capecitabine is a fluoropyrimidine carbamate that was rationally designed as an orally administered precursor of 5′-deoxy-5-fluorouridine, which is selectively tumor-activated to the cytotoxic agent 5-FU by exploiting the higher levels of thymidine phosphorylase found in tumor tissues compared with normal tissues [6]. Capecitabine is converted to 5-FU preferentially in tumor tissue via a three-step enzymatic cascade, firstly converted to 5′-deoxy-fluorocytidine by hepatic carboxylesterase in the liver; secondly converted to 5′-deoxy-5-fluorouridine by cytidine deaminase in the liver and tumor tissues; finally converted to 5-FU at the tumor site by the tumor-associated angiogenic factor thymidine phosphorylase, thereby minimizing the exposure of normal tissue to 5-FU [18]–[20]. In phase II studies, the main toxicities were hand-foot syndrome and diarrhea. The pCR rate was ranging from 12% to 31% and overall downstaging rate was ranging from 59% to 84% [21]–[23]. The short-term outcomes after preoperative therapy, such as tumor volume reduction, pathological downstaging, toxicity, were similar to 5-FU infusion [7].

S-1 is an oral anticancer drug that combines tegafur (a prodrug that is converted by cells to 5-FU), 5-chloro-2,4-dihydropyrimidine (CDHP) and potassium oxonate in a molar ratio of 1∶0.4∶1 [8], [24]. CDHP is a potent and reversible inhibitor of dihydropyrimidine, thereby prolonging high 5-FU concentration in the circulation [25], [26]. Ptassium oxonate is a inhibitor of orotate phosphoribosyltransferase that catalyzes the phosphorylation of 5-FU in the gastrointestinal tract, thereby reducing the gastrointestinal toxic effects of 5-FU [27]. Nakata et al. [28] reported that S-1 could enhance the radiation response of human colon cancer xenografts resistant to 5-FU. In a Phase II study, the rates of pCR, overall downstaging, tumor volume reduction were 22%, 78%, 69±22%, respectively. The adverse events were mild and hand-foot syndrome was not observed [9].

As mentioned above, both S-1 and capecitabine have been used in preoperative CRT for LARC patients, but lack direct comparison between them. According to our knowledge, this is the first study to compare efficacy and safety in patients treated with capecitabine or S-1 in preoperative CRT for LARC using a retrospective matched-pair analysis. In this study, both S-1 group and capecitabine group achieved a high efficacy of tumor responses, and patients in either group could tolerate the treatment-related adverse events. The treatment compliance was extremely high in two groups and all the patients completed the treatment schedule, neither interruption nor dose reduction (including chemotherapy drugs and radiation). Comparison of S-1 group and capecitabine group, the tumor responses such as tumor volume reduction, downstaging and tumor regression did not differ significantly. However, patients treated with capecitabine suffered more adverse events than who treated with S-1. Diarrhea and hand-foot syndrome were mainly discovered in capecitabine group. Thus, patients who treated with capecitabine should pay more money on managing adverse events.

The present study had some limitations. First, the follow-up time was short. Only short-term outcomes were assessed in this study. Overall survival and disease free survival data, as long-term outcomes, need further study to analyze between the two regimens. Second, the number of cases was small and clinical significance was limited. The next step will be to further expand the number of cases. Third, this was a retrospective study. It is advisable to conduct a multicenter randomized controlled study.

## Conclusions

The both preoperative CRT regimens were effective and safe for patients of LARC. However, regimen with S-1 had a lower incidence of adverse events. Thus, this retrospective matched-pair study suggested that preoperative CRT with S-1 is a more reasonable choice for LARC.

## Supporting Information

Table S1 **Baseline characteristics of the rectal cancer patients in two group.**
(XLSX)

Table S2 **The clinical response and pathological response of the rectal cancer patients in two group.**
(XLSX)

Table S3 **The treatment-related adverse events of the rectal cancer patients in two group.**
(XLSX)

Table S4 **The clinical stage after preoperative chemoradiotherapy of the rectal cancer patients in two group.**
(XLSX)
